# Does the change between the native and the prosthetic posterior tibial slope influence the clinical outcomes after posterior stabilized TKA? A review of 793 knees at a minimum of 5 years follow-up

**DOI:** 10.1051/sicotj/2025014

**Published:** 2025-03-27

**Authors:** Hassan Alhamdi, Etienne Deroche, Jobe Shatrov, Cécile Batailler, Sébastien Lustig, Elvire Servien

**Affiliations:** 1 Orthopaedic Surgery and Sports Medicine Department, FIFA Medical Center of Excellence, Croix-Rousse Hospital, Lyon University Hospital 103 grande rue de la Croix Rousse 69004 Lyon France; 2 Sydney Orthopaedic Research Institute, University of Notre Dame Australia, Hornsby and Ku-Ring Hospital Palmerston Rd Hornsby NSW 2077 Sydney Australia; 3 University of Lyon, Claude Bernard Lyon 1 University, IFSTTAR, LBMC UMR_T9406 25 Av. François Mitterrand 69500 Bron France; 4 LIBM – EA 7424, Interuniversity Laboratory of Biology of Mobility, Claude Bernard Lyon 1 University 4 Rue Raphaël Dubois 69100 Villeurbanne France

**Keywords:** Total knee arthroplasty, Posterior-stabilized, Tibial slope, Knee flexion, Survivorship

## Abstract

*Introduction*: The understanding of the influence of posterior tibial slope (PTS) on knee kinematics has increased. However, the PTS influence on clinical outcomes remains unclear. The study aimed to evaluate whether a significant change between the native and the prosthetic tibial plateau PTS influences functional results and the risk of complications following total knee arthroplasty (TKA). *Methods*: This was a retrospective, monocentric comparative study. Clinical and radiological data from 793 knees were collected from a prospective surgical database. Inclusion criteria were patients operated with a posterior-stabilized TKA (PS-TKA) for primary tibiofemoral osteoarthritis, with or without associated patellofemoral osteoarthritis, or osteonecrosis of the femoral condyle or tibial plateau, with a minimum follow-up of 5 years. Range of motion and International Knee Society (IKS) score as well as radiological measurements were collected preoperatively and postoperatively at each follow-up visit. Two groups were composed according to the change in PTS between pre- and post-op (Group 1: ≤10°, *n* = 703; Group 2: >10°, *n* = 90). *Results*: The mean follow-up was 75.5 months ± 9.1. The mean change in PTS from preoperative was 4.96° ± 3.24 in group 1 and 12.7° ± 1.87 in group 2. There was no significant difference in the mean IKS Knee subscore (89.5 ± 10.7 and 89.7 ± 10.2, *p* = 0.89) and mean IKS Function subscore (88.2 ± 15.7 and 86.3 ± 16.6, *p* = 0.33) in groups 1 and 2, respectively. Postoperative maximum flexion was very satisfactory in both groups with no clinically relevant difference (120.0 ± 11.9 and 123.0 ± 8.3, *p* = 0.026). The complication rate was 5.0% (*n* = 40) (5.5% in group 1; 1.1% in group 2; *p* = 0.07) while the most common complication requiring further procedure was deep infection (*n* = 9, 1.1%) and the second most common was stiffness (*n* = 6, 0.8%). *Discussion*: PTS did not influence postoperative maximum flexion or clinical scores and was not associated with a higher complication rate at a minimum 5-year follow-up after PS-TKA.

## Introduction

Total knee arthroplasty (TKA) is an effective procedure for relieving pain and restoring function in patients with end-stage knee osteoarthritis [1]. While multiple factors contribute to the success of TKA, the role of anatomical variations (coronal, sagittal, and axial alignments) in influencing postoperative outcomes has garnered increasing attention.

The posterior tibial slope (PTS) is the slope of the tibial plateau relative to the longitudinal axis of the tibia and has been implicated in altering knee biomechanics and potentially impacting the functional and clinical results of TKA [2, 3]. It has an indirect role in flexion stability and flexion gap, facilitates femoral rollback and may promote a greater range of motion (ROM). Several biomechanical studies have shown that PTS is positively correlated with the range of flexion [4–6].

The influence of PTS on the ROM or functional outcomes after cruciate-retaining TKA (CR-TKA) [7–9] or posterior-stabilized TKA (PS-TKA) [10–12] has been studied but remains unclear. While some studies propose that modifying tibial slope during TKA can lead to improved kinematics and clinical outcomes [5, 13], others argue that such alterations might introduce an increase in the risk of complications such as implant loosening, instability, and uneven load distribution [7, 10]. Furthermore, follow-up has either been short or limited to small cohorts. There is very limited evidence of the impact of postoperative PTS change on the functional outcome of PS-TKA before 10 years of follow-up.

This study aims to assess the influence of the modification in PTS on clinical outcomes (International Knee Society Score, range of motion, complications, and revisions). We hypothesised that PTS modification of less than 10° would obtain better functional outcomes compared to deviations of than 10°.

## Materials and methods

### Study design

This retrospective comparative study was conducted at a single institution. Patients who underwent TKA from 1987 to 2016 and received one of two types of PS-TKA implants were identified from a prospective institutional database. A total of 4745 patients were identified, 4116 patients (TORNIER-CORIN^©^, Montbonnot Saint Martin 38330, France) and 629 patients (AMPLITUDE^®^, Valence 26000, France).

### Patient selection

**Figure 1:**
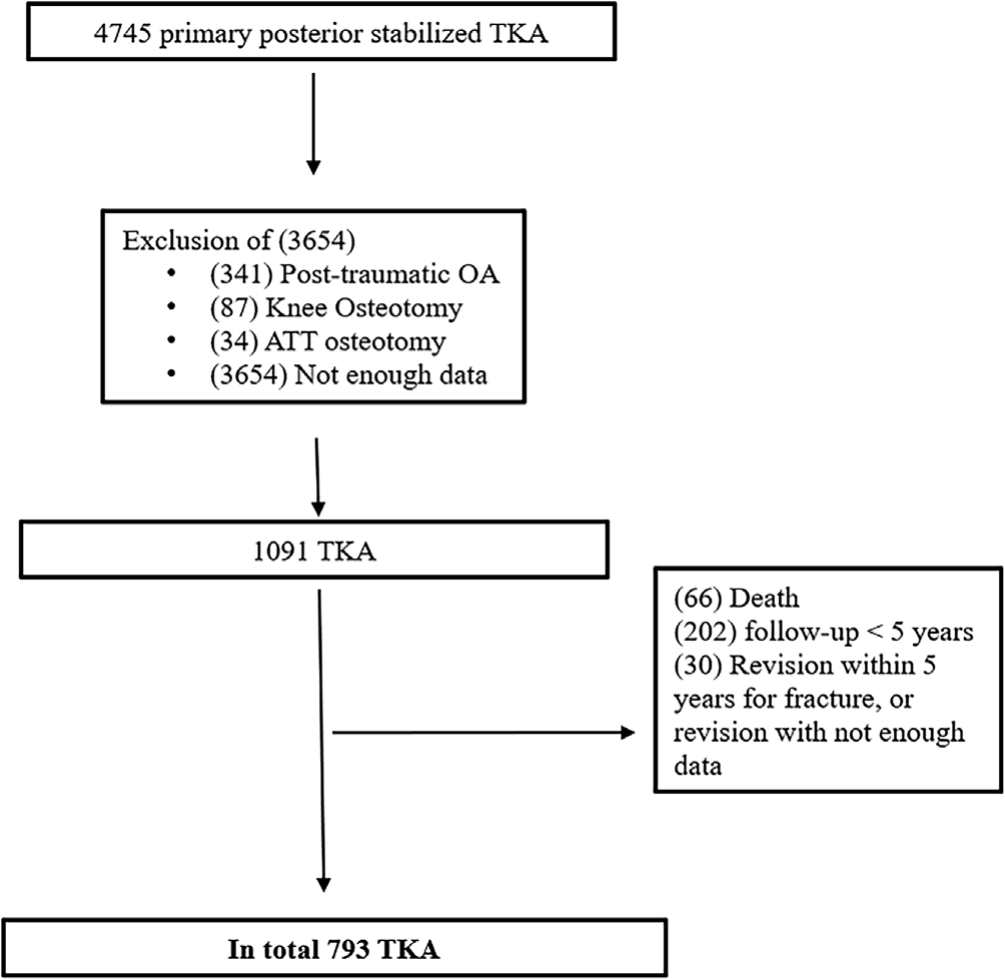
Flowchart.

Inclusion criteria were patients aged over 18 requiring a primary TKA for knee OA or suffering from osteonecrosis of the femoral condyle or tibial plateau. Exclusion criteria were post-traumatic OA, inflammatory arthritis, history of osteotomy, need for tibial tubercle osteotomy (TTO) during the surgery, and patients with a follow-up of less than 5 years. A total of 793 knees were eligible for inclusion (Figure 1). Clinical outcomes were analyzed and divided into two groups according to the modification of PTS (modification by ≤10° in Group 1 and >10° in Group 2). PTS modification was calculated by subtracting the preoperative value from the postoperative value.

Seven hundred and ninety-three patients were included in the final analysis. For the analysis, patients were divided into two groups according to the amount of PTS deviation from their native state. In group 1 (≤10°), there were 703 knees; in group 2, there were 90 knees. In the total cohort, the mean age at the index procedure was 69.5 ± 8.46 years and the mean follow-up was 75.5 ± 9.1 months (min = 60; max = 216). Patient demographic information between the two groups is presented in Table 1.

**Table 1:** The demographic data between the two groups.

	Group ≤ 10 (*n* = 703)	Group > 10 (*n* = 90)	*P* value
Sex, *n*			
Women	366 (52%)	41 (46%)	0.3
Men	337 (48%)	49 (54%)	–
Side, *n*			
Left	353 (50%)	45 (50%)	0.98
Right	350 (50%)	45 (50%)	–
Age (years), Mean ± SD	69.4 ± 8.5	70.5 ± 8.2	0.22
Min/Max	41/95	50/84	
Height (cm), Mean ± SD	165.0 ± 9.4	166.0 ± 9.2	0.62
Min/Max	130/192	145/187	
Weight (kg), Mean ± SD	79.8 ± 16.2	80.0 ± 14.5	0.99
Min/Max	41/166	54/125	
BMI (kg/m^2^), Mean ± SD	29.3 ± 5.5	29.1 ± 4.9	0.76
Min/Max	160/542	193/437	
Follow-up (months), Mean ± SD	75.3 ± 8.2	75.8 ± 10.0	0.26
Min/Max	60/170	60/216	
Implant (KneeTec) (%)	457 (65%)	59 (66%)	1

BMI: Body mass index, SD: standard deviation

### Surgical procedure and rehabilitation

All operations were performed using the same surgical technique, by three experimented surgeons with similar training. All patients received a PS-TKA: Either an Anatomic (AMPLITUDE^®^, Valence 26000, France) or Kneetec (TORNIER-CORIN^©^, Montbonnot Saint Martin 38330, France). A trans-quadriceps tendon approach was performed for all patients (medial approach for all varus; lateral approach for all valgus knees as it was described by Verdonk et al. [14] and Gunst et al. [15]). The target postoperative mechanical alignment was neutral with a Hip Knee Ankle (HKA) angle at 180°. It was a measured resection technique with releases, with a goal of symmetric coronal balance. A combination of intramedullary and extramedullary guides was used for the primary tibial cut. An intramedullary guide was used to perform the distal femoral cut with a valgus of 7° in the case of varus deformity and 5° in the case of valgus deformity. In cases of lateral condyle hypoplasia, the femoral component was positioned with 3° of external rotation. Posterior referencing was used to size the femoral component. The tibia was cut with 0° of posterior slope according to the intramedullary guide. Each implant has a built-in posterior slope; 3° in the Anatomic, AMPLITUDE^®^, and 2.3° in the Kneetec, TORNIER-CORIN^©^ [16, 17]. Patellar resurfacing was performed in 83.3% of cases (661 knees) according to anterior knee pain and patellofemoral osteoarthritis. A standard post-operative physical therapy regimen was initiated following surgery focusing on regaining knee ROM, immediate full weight-bearing and quadriceps strengthening. Manipulation under anaesthesia was performed in patients who failed to progressively regain motion (>90°) with therapy by 8 weeks post-operatively. Arthroscopic arthrolysis was performed in cases of persistent stiffness in flexion, defined by a flexion inferior to 90° after 2 months postoperatively.

### Evaluation and outcome variable

Preoperative patient demographics (age, gender, side, height, weight, and body mass index) were collected as well as preoperative and postoperative passive ROM and International Knee Society Score (IKS knee and function) [18]. The patients had a systematic follow-up at 2 months, 1 year, 2 years, 5 years, and every 5 years.

All complications were collected at the last follow-up. A complication was defined as an intercurrent event compromising the functional and vital status and requiring a new consultation or hospitalization.

**Figure 2:**
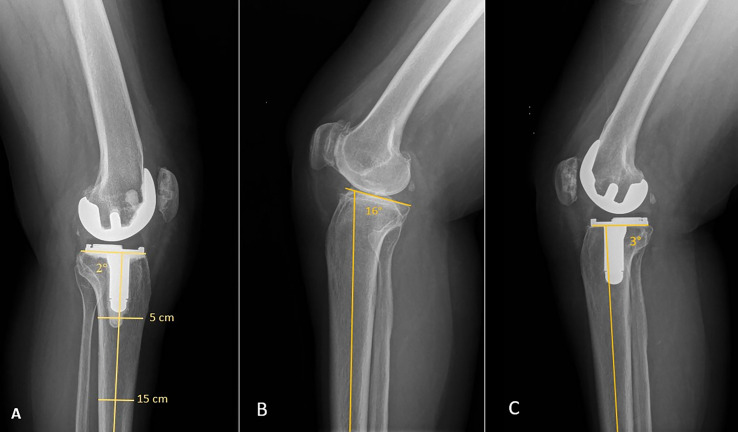
(A) Technique for measuring posterior tibial slope. Two lines are drawn: line 1 follows the native lateral tibial plateau or the prosthetic tibial baseplate; and line 2 connects two points located 5 cm and 15 cm distal to the joint line, midway between the anterior and posterior tibial cortex. The posterior tibial slope was measured as the angle between line 1 and the perpendicular line to line 2. (B) and (C) Case of a change in posterior tibial slope greater than 10°.

Radiological measurements were performed preoperatively and postoperatively at each follow-up by a senior surgeon. Digital radiographs were obtained according to the Digital Imaging and Communications in Medicine (DICOM) standards using a calibrated magnification. For each patient, the HKA angle, the femoral and tibial mechanical axis as well and the PTS were measured by an independent orthopaedic surgeon, with an accuracy of 0.1°. On lateral knee radiographs, the longitudinal component of the PTS measurement was determined using a previously described measurement technique that defines the proximal anatomic axis of the tibia on lateral X-ray view [19, 20]. Two lines are drawn: line 1 follows the native lateral tibial plateau (for preoperative measurements) or the prosthetic tibial baseplate (for postoperative measurements); and line 2 connects two points located 5 cm and 15 cm distal to the joint line, midway between the anterior and posterior tibial cortex. The PTS was measured as the angle between line 1 and the perpendicular line to line 2 and subtracted from 90° which gave the value of PTS (Figure 2).

#### Data analyses

Statistical analysis performed with Medistica^©^, pvalue.io (*graphic user interface to the R statistical analysis software 2019–22).* Continuous variables were expressed as their mean and standard deviation. Descriptive data were analyzed using the Student’s *t*-test for non-categorical variables and the Pearson χ^2^-test for categorical variables. A *p-*value of <0.05 was accepted as statistical significance. Survival analysis was performed by the Kaplan-Meier method, with any implant revision as endpoints.

## Results

The IKS score rose from 123 ± 27.5 before surgery to 177 ± 22.5 at the last follow-up, with a statistically significant improvement at mid-term follow-up in both groups. The mean PTS changed from 7.4 ± 3.86° to 1.6 ± 1.7° (*p* < 0.001).

Preoperative and postoperative functional scores and radiological analysis between the two groups are shown in Table 2. The groups were similar for all preoperative characteristics except for the mean PTS (6.6 ± 3.2 vs. 13.6 ± 2.4 in groups 1 and 2 respectively, *p* < 0.001). There was a statistically significant difference in the mean postoperative PTS (1.6° ± 1.7 vs. 0.9° ± 1.8 in groups 1 and 2 respectively, p < 0.001), but the difference was not clinically relevant.

**Table 2:** Preoperative and postoperative clinical and radiological assessment between both groups.

	Preoperative			Postoperative		
	Group ≤ 10° (*n* = 703)	Group > 10° (*n* = 90)	*P* value	Group ≤ 10° (*n* = 703)	Group > 10° (*n* = 90)	*P* value
	Mean ± SD	Mean ± SD		Mean ± SD	Mean ± SD	
	Min/Max	Min/Max		Min/Max	Min/Max	
Flexion	119.0 ± 14.7	118.0 ± 14.4	0.54	120.0 ± 11.9	123.0 ± 8.3	0.026
	60/140	70/140		70/140	70/140	
Knee IKS score	60.2 ± 16.0	57.1 ± 17.0	0.1	89.5 ± 10.7	89.7 ± 10.2	0.89
	9/93	20/86		24/100	39/100	
Function IKS score	62.6 ± 15.6	64.4 ± 15.1	0.28	88.2 ± 15.7	86.3 ± 16.6	0.33
	10/100	20/95		20/100	20/100	
Total IKS score	123.0 ± 27.5	122.0 ± 28.1	0.66	177.0 ± 22.5	176.0 ± 22.4	0.69
	36/193	84/200		69/200	84/200	
HKA	176.0 ± 7.4	175.0 ± 8.5	0.11	179.0 ± 2.6	179.0 ± 2.7	0.47
	158/202	162/202		156/186	172/186	
mFA°	91.6 ± 3.29	91.1 ± 3.61	0.21	89.8 ± 1.9	89.6 ± 1.9	0.29
	83/103	82/101		82/98	85/96	
mTA°	86.7 ± 5.8	86.6 ± 4.2	0.87	89.2 ± 1.8	89.3 ± 1.8	0.63
	75/102	76/97		79/95	82/94	
PTS	6.6 ± 3.2	13.6 ± 2.4	<0.001	1.6 ± 1.6	0.9 ± 1.8	<0.001
	−16/15	8/21		−3/7	−5/7	
Slope difference				4.96 ± 3.2	12.7 ± 1.8	<0.001
				−16/10	11/19	

HKA°: Hip Knee ankle angle, mFA°: mechanical femoral frontal angle, mTA°: mechanical tibial frontal angle, IKS: International Knee Society score, PTS: Posterior tibial slope, SD: standard deviation.

There was no significant difference in mean IKS Knee subscore (89.5 ± 10.7 and 89.7 ± 10.2, *p* = 0.89) and IKS Function subscore (88.2 ± 15.7 and 86.3 ± 16.6, *p* = 0.33) in groups 1 and 2 respectively. Postoperative maximum flexion was satisfactory in both groups with no clinically relevant difference (120° ± 11.9° and 123° ± 8.3°, p = 0.026). While there is a statistically significant difference between the two groups, the difference of 3° is not clinically relevant.

**Figure 3:**
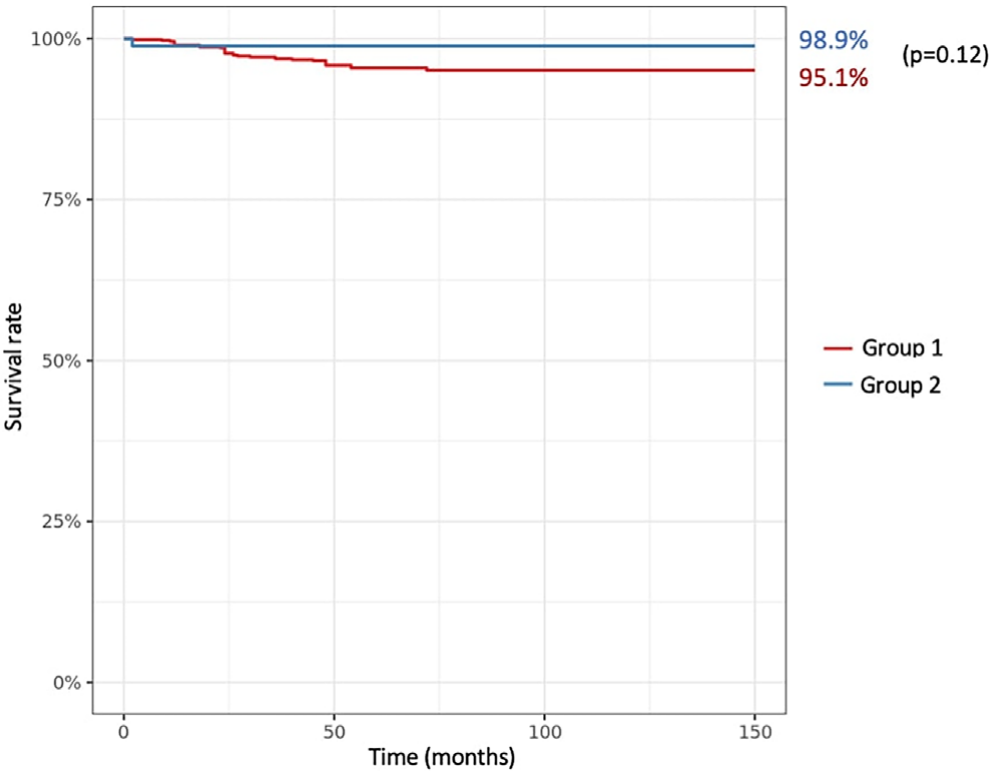
Survival analysis in both groups according to Kaplan Meier, with any implant revision as endpoints. Red curve: the group where PTS change ≤ 10°. Blue curve: the group where PTS change of > 10°.

The overall complication rate was 5.0% (*n* = 40) as shown in Table 3 (5.5% in group 1; 1.1% in group 2; *p* = 0.07). There were 34 patients (4.3%) requiring an additional procedure (group 1: *n* = 33; group 2, *n* = 1), and for 16 patients (2.0%) a revision of the femoral and/or tibial component was necessary (all in group 1). The overall survival rate of the total cohort was 95.5% (CI95% 94.0–97.0), and 95.1% (CI95% 93.4–96.8) in group 1 versus 98.9% (CI95% 96.7–100.0) in group 2 (*p* = 0.12) (Figure 3). The most common cause of reoperation was deep infection (*n* = 9, 1.1%) followed by stiffness (*n* = 6, 0.8%) and aseptic loosening (*n* = 5, 0.6%). There were seven cases of loosening, one for septic reason (femoral component) and six considered as aseptic, including three tibial loosening, and three patellar buttons loosening.

**Table 3:** Complications in both groups.

	Group ≤ 10° (*n* = 703)	Group > 10° (*n* = 90)	*P* value
All complication	39 (5.5%)	1 (1.1%)	0.07
Revision for all causes	33 (4.7%)	1 (1.1%)	0.12
Femoral loosening	1 (0.1%)		0.72
Tibial loosening	3 (0.4%)		0.53
Patellar button loosening	3 (0.4%)		0.53
Patellar resurfacing	4 (0.6%)		0.47
Patellar instability	4 (0.6%)		0.47
Lateral patellar facetectomy	3 (0.4%)		0.53
Infection	8 (1.3%)	1 (1.1%)	0.97
Stiffness	2 (0.3%)		0.61
Laxity	2 (0.3%)		0.61
Skin necrosis	1 (0.1%)		0.72
Clunk syndrome	1 (0.1%)		0.72
Oversizing	1 (0.1%)		0.72
Complications not requiring surgical revision	6 (0.8%)		0.38
Stiffness (mobilization under anesthesia)	4 (0.6%)		0.47
CRPS	2 (0.3%)		0.61

CPRS: complex regional pain syndrome.

## Discussion

There is abundant but conflicting literature on the relationship between tibial slope and maximum flexion achieved after PS-TKA. However, to the best of our knowledge, no previous studies have evaluated the clinical consequences of altering the native tibial slope. Contrary to our hypothesis, a change of more than 10° in the native tibial slope with a PS prosthesis did not have a deleterious effect on the medium-term results.

A wide range of native PTS has been reported in patients with knee OA. Hofmann et al. have reported that the normal PTS is 7° [21], between 8°−10° by Laskin and Rieger [22], and 14° by Chiu et al. [23]. Studies have reported differences of up to 5°, and interindividual (medial and lateral posterior tibial slopes) ranges as high as 30° [24]. Despite these observations, the clinical consequences of deviations from the native state following TKA remain unclear.

The correct target for setting the posterior slope in TKAs remains debated. Both the choice of implant design and alignment philosophy affect the PTS slope target. In primary TKA the target PTS varies between 3° and 7° [24]. In PS-TKA, a PTS between 0° and 3° is typically recommended to provide tibial component stability and avoid excessive posterior compressive strains and flexion instability, and these guidelines were followed in our population [25]. With cruciate-retaining implant design, an angle up to 5°–7° can be aimed to achieve adequate ROM, particularly in flexion [26].

The difference between the two groups in postoperative maximum flexion was statistically significant (120.0 ± 11.9 and 123.0 ± 8.3, *p* = 0.026). However, the difference of 3° is not clinically relevant in this study. Radhamony et al. [27] showed that a change in the PTS does not affect the postoperative ROM in PS-TKR using navigation assists used in their population of 120 knees. They were divided into three groups according to the difference in PTS (Group 1: 0–7.5; Group 2: 7.6–15 and Group 3: 15.1–22.5). The mean difference in the PTS in the three groups was 4.5°, 10.8°, and 18.0°, and there was no statistically significant change in ROM with that of change in PTS. Another prospective randomized controlled study with 31 consecutive patients underwent TKA using a cutting block and intramedullary cutting guide designed to impart a 0-degree posterior tibial slope (group 1) and a 5-degree tibia cutting block was used in 30 subsequent patients (group 2) with a posterior cruciate–sacrificing design. With a minimum 3-month follow-up, they revealed that PTS did not have any effect on post-operative maximal flexion or Hospital for Special Surgery functional score [10]; with a mean postoperative flexion was 123.3° in group 1 (range: 90°–135°) and 119.6° in group 2 (range: 75°–135°). However, they did not exactly analyze the influences of the modification of PTS from the native one, and they focused only on the very short term. Only one retrospective cohort study with long-term (mean follow-up of 9 years) reported the impact of the PTS on postoperative flexion. They evaluated 65 knees who underwent TKA with a PS prosthesis and were divided into the following three groups according to the measured tibial slopes in postoperative: Group 1: ≤4°, Group 2: 4°–7° and Group 3: >7° [12]. They demonstrated a positive significant difference in increasing PTS regarding the maximal flexion in PS-TKA [12]. They found that an increase of 1° of PTS can lead to 1.8° greater flexion.

This study did not show any correlation between PTS change and IKS knee or function scores at the last follow-up. These results are similar to the literature. Kansara and Markel [10] did not find a correlation between PTS change and Hospital for Special Surgery functional score at a minimum of 3 months follow-up in a prospective randomized controlled study. The design of knee prosthesis (PCL-retaining vs. PCL-sacrificing) is an important factor that should be considered in evaluating the relationship between PTS and TKA outcomes. Catani et al. [4] have conducted a biomechanical study showing that PTS is positively correlated with the range of flexion and inhibits excessive tension in the posterior cruciate ligament (PCL) during knee flexion. The fear of excessive PTS is to lead to anterior dislocation of the tibia and biomechanical changes of the knee, compromising the longevity of TKA.

Contrary to our hypothesis, there was no statistically clinically significant difference in comparison between pre- and postoperative modification in ROM, functional score, and any correlation regarding complication among the two groups.

The main limitations of this study include its retrospective design on a large period of inclusion, with a mechanical alignment and measured resection technique. However, this study has the merit of being homogeneous and the largest we are aware of, including a focused analysis of the modification of PTS in PS-TKA at a minimum follow-up of 5 years. One of the limitations of our study is that factors such as the posterior condylar offset were not taken into consideration, which can affect the postoperative ROM. Another limitation is the large difference in sample size between the two groups. Nevertheless, a better understanding of the effect of tibial slope changes in PS-TKA is warranted. Nonetheless, a reduction in PTS may also lead to a decrease in the flexion ROM of the knee [12]. Hence, the degree of reduction in the PTS and its impact on postoperative outcomes deserves further prospective comparative studies with large populations to confirm these results. Then, no power analysis was performed at the beginning of the study.

## Conclusion

PTS did not influence postoperative maximum flexion or clinical scores and was not associated with a higher complication rate at a minimum 5-year follow-up after PS-TKA.

## Data Availability

Data are not available.
